# Manufacture of Carbon Materials with High Nitrogen Content

**DOI:** 10.3390/ma15072415

**Published:** 2022-03-25

**Authors:** David Villalgordo-Hernández, Aida Grau-Atienza, Antonio A. García-Marín, Enrique V. Ramos-Fernández, Javier Narciso

**Affiliations:** 1 Laboratorio de Materiales Avanzados, Departamento de Química Inorgánica, Instituto Universitario de Materiales de Alicante, Universidad de Alicante, Apartado 99, 03080 Alicante, Spain; dvh5@gcloud.ua.es (D.V.-H.); aida.grau2@gmail.com (A.G.-A.); antoniocaravaca95@gmail.com (A.A.G.-M.); enrique.ramos@ua.es (E.V.R.-F.); 2 Instituto de Investigación Sanitaria y Biomédica de Alicante (ISABIAL), 03010 Alicante, Spain

**Keywords:** activated carbon, CO_2_ adsorption, N-doped porous carbon

## Abstract

Nowadays one of the biggest challenges for carbon materials is their use in CO_2_ capture and their use as electrocatalysts in the oxygen reduction reaction (ORR). In both cases, it is necessary to dope the carbon with nitrogen species. Conventional methods to prepare nitrogen doped carbons such as melamine carbonization or NH_3_ treatment generate nitrogen doped carbons with insufficient nitrogen content. In the present research, a series of activated carbons derived from MOFs (ZIF-8, ZIF-67) are presented. Activated carbons have been prepared in a single step, by pyrolysis of the MOF in an inert atmosphere, between 600 and 1000 °C. The carbons have a nitrogen content up to 20 at.% and a surface area up to 1000 m^2^/g. The presence of this nitrogen as pyridine or pyrrolic groups, and as quaternary nitrogen are responsible for the great adsorption capacity of CO_2_, especially the first two. The presence of Zn and Co generates very different carbonaceous structures. Zn generates a greater porosity development, which makes the doped carbons ideal for CO_2_ capture. Co generates more graphitized doped carbons, which make them suitable for their use in electrochemistry.

## 1. Introduction

Currently the use of carbon materials has grown greatly. Its use has spread especially in electrochemistry and as a possible solution in reducing the greenhouse effect by capturing CO_2_. In both cases the presence of N in the structure is considered as essential. Recently Cazorla and co-workers [1] have published a review on the presence and importance of heteroatoms in carbon materials in their use as electrodes in ORR (oxygen reduction reaction), where it has been shown that the presence of N is essential, especially pyridine species and quaternary nitrogen. These authors have carried out different works within the area, where the sample made from the use of a hard template (Y zeolite) [2] based on the original work by Kyotani [3] but using acetonitrile as a C precursor is remarkable in order to introduce N in the structure. However, the maximum content of nitrogen is below 4 at.%. Nevertheless, they found a very high improvement in its use as a supercapacitor. Other authors in their search for increasing the CO_2_ adsorption capacity have carbonized polyaniline [4] where carbon materials with a nitrogen content up to 8 at.% do not seem to have a fundamental role in CO_2_ capture, but rather the capacity depends mainly on the ultramicroporous content (<0.6 nm). In principle, this partially contradicts previous studies where they conclude that the presence of amines improves CO_2_ adsorption, although the main factor remains the microporosity of the sample [5]. However, some authors have reported that the presence of nitrogen does favor CO_2_ capture [6,7], but their synthetic method is completely different. Some authors have developed other types of strategies to generate carbons doped with nitrogen, basically using a ball mill, where they jointly grind a carbon precursor such as graphene oxide and melamine, also synthesize C_3_N_4_ in a similar way, where they show that the new generated material ostensibly improves the catalytic properties in the ORR [8,9,10]. We can conclude that the presence of N is necessary, but only once what type of N is adequate for the capture to be more efficient has been determined. On the other hand, it is necessary to develop new synthetic methods that increase the N content, since those described above only achieve content lower than 8 at.%.

The main competitor of carbon in CO_2_ capture are MOFs (Metallic Organic Frame-works), since there is great versatility in both porosity and surface chemistry [11,12]. However, many of them are unstable under the application conditions. One of the most interesting is UiO-66 [13] due to its easy manufacture and great stability, although it is not the most suitable for CO_2_ capture. Modification of the linker (terephthalic acid by 2-aminoterephthalic acid) increases its adsorption capacity significantly [14] making it comparable to the best CO_2_ absorbents. This result highlights the fact that the presence of N is essential in the capture of CO_2_. ZIFs (Zeolitic Imidazolate Frameworks) are composed of tetrahedrally-coordinated transition metal ions (e.g., Fe, Co, Cu, Zn) connected by imidazolate linkers. ZIF is topologically isomorphic with zeolite because the Si-O-Si bond angles are equal to M-N-M. ZIF-8 and ZIF-67 are two isostructural MOFs whose formula is M(Imz)2, where Imz is 2-Methylimidazole, and M is the divalent cation. The structure it presents is the same as zeolite-A, and from this derives its great stability. As we have commented on some MOFs, especially ZIF have great stability since their decomposition temperature in a non-oxidizing atmosphere is close to 500 °C, which implies that it could generate carbons with a high N content. This fact has not been overlooked and various authors have obtained carbon materials by pyrolysis of ZIF-8, one of the most popular MOFs due to its easy synthesis and great robustness [15,16].

This manuscript presents a series of carbons obtained from ZIF-8 and ZIF-67, and some derivatives of them, where it is shown that it is possible to obtain carbons with a N content greater than 20 at.% for its application in CO_2_ capture, catalysis and electrocatalysis.

## 2. Materials and Methods

The synthesis followed the established processes described elsewhere [17,18]. In a typical synthesis 144 mmol of 2-Methylimidazole was dissolved in 50 mL of deionized water, 12 mmol of cobalt or zinc acetate was dissolved in 25 mL of deionized water. Then, both solutions were blended and stirred vigorously during 5 min and kept for 72 h at room temperature, after which the precipitated MOF was separated by centrifugation and washed 3 times with MeOH. Finally, the MOF powder was dried for 24 h at 60 °C in a conventional oven. All chemicals have been provided by Aldrich, Mollet del Vallès, Barcelona, Spain (analytical grade) and have been used without further purification. Once of MOF was prepared, the effect of carbonization temperature was studied. The MOFs were carbonized for 2 h in a tubular furnace (Carbolite STF/450, UK) under dynamic atmosphere (100 mL/min N_2_), at 5 °C/min from 600 to 1000 °C. The samples were labeled as MOFC_T, where T indicates the carbonization temperature. After carbonization the carbon material was washed with HCl following the procedure of Gascón [19], (i.e., ZIF67C_700_2l means activated carbon obtained by carbonization of ZIF67 at 700 °C and washed 2 times with HCl).

### Characterization

For the study of porosity, the Quantachrome device (Quadrawin, Boynton Beach, Florida, USA has been used. The study has been carried out by adsorption-desorption of nitrogen at 77 K and carbon dioxide at 273 K. The surface area is determined from the branch of adsorption of the nitrogen isotherm applying the BET equation, for the analysis a minimum of 5 points have been used, and in all cases the C is positive [20]. Samples were outgassed at 150 °C for 4 h prior to the adsorption measurements. The minimum area that can be analyzed is 1 m^2^/g. The accuracy is higher than 2% for areas greater than 100 m^2^/g. Micropore Volume (V_micro_) was estimated by the Dubinin-Raduskevich (DR) method. Pore Volume has been obtained at P/P_0_ = 0.95. The objective of CO_2_ adsorption is to determine if there is any diffusional restriction, obtaining the volume of micropores from the DR equation. No carbon sample shown any diffusional restrictions.

A Bruker (Billerica, Massachusetts, USA) powder diffractometer (D-8 Advanced Diffractometer) has been used to determine the crystalline phases, using Cu radiation with a Ni filter and a Goëbel mirror. The X-ray generation system is the Kristalloflex K-760 80F. Diffractograms were registered between 5° and 80° with a step of 0.05° and a step time of 3 s.

VG-Microtech Multilab device (VG-Microtech, London, UK) with Mg Kalpha (Hv: 1253.6 eV) radiation source, pass energy of 50 eV and background pressure of 5 × 10^−7^ Pa has been used to XPS measurements. A gingerly deconvolution of the spectra has been carried out, and the areas of the peaks were obtained by calculating the integral of each peak after subtracting a Shirley background and fitting the experimental peak to a combination of Lorentzian/Gaussian in a proportion 30/70.

A Bruker (Billerica, Massachusetts, USA) Field Emission Scanning Electron Microscopy with X-ray microanalysis (SEM–EDS) (ZEISS-Merlin VP Compact, BRUKER-Quantax 400) has been used to study the morphology of the carbon materials in both Backscattered Electron (BSE) and Secondary Electron (SE) modes. An also from Bruker, Spain Transmission Electron Microscopy (TEM) (JEOL-JEM-1400 Plus) has been used in some samples.

TG-DTA-MS has been carried out in TGA/SDTA851e/LF/1600 from Mettler Toledo, (Columbus, OH, USA) equipped with the Thermostar GSD301T Pfeiffer mass spectrometer. The TG experiments were carried out in the dynamic atmosphere of Ar (100 cm^3^/min), and in a dynamic O_2_/Ar (1/4) atmosphere; with a heating rate of 10 °C/min, while scanning masses up to 200 amu. The accuracy is higher of 1%.

## 3. Results and Discussion

**Figure 1:**
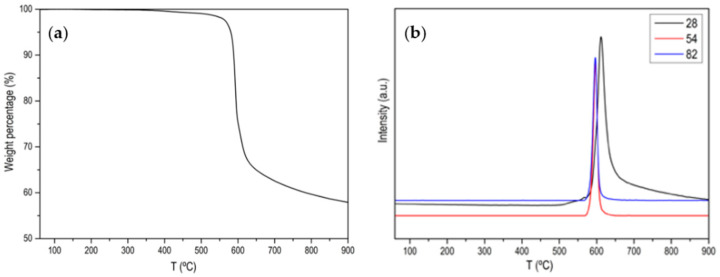
(**a**) Normalized TG profile of ZIF-67 in Ar atmosphere; (**b**) MS signal for *m*/*z* 28 (N_2_), *m*/*z* 54 and *m*/*z* 82 (main 2-Methylimidazole fragmentations).

**Figure 2:**
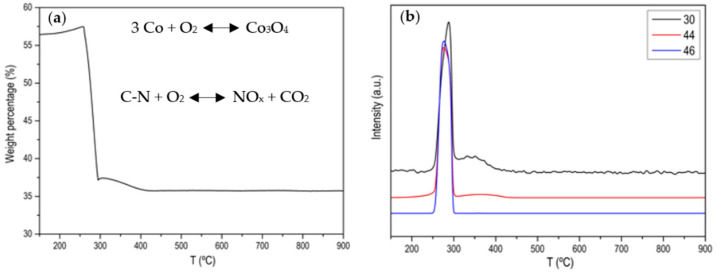
(**a**) Normalized TG profile of ZIF-67 in air atmosphere after the treatment in Ar atmosphere; (**b**) MS signal for *m*/*z* 30 (NO), *m*/*z* 46 (NO_2_) and *m*/*z* 44 (CO_2_).

**Figure 3:**
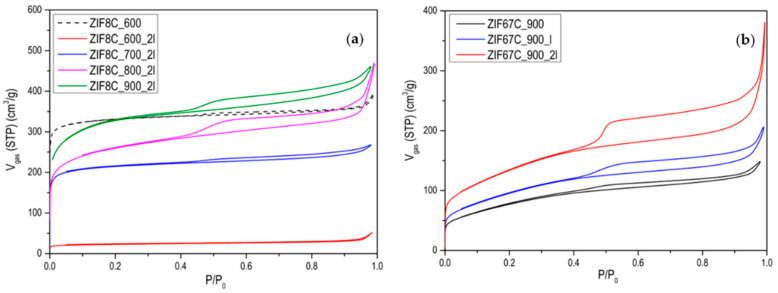
N_2_ adsorption isotherms at −196 °C of (**a**) the activated carbons obtained from ZIF-8; (**b**) the activated carbons obtained from ZIF-67 at 900 °C, and the effect of the washing with HCl.

**Figure A1:**
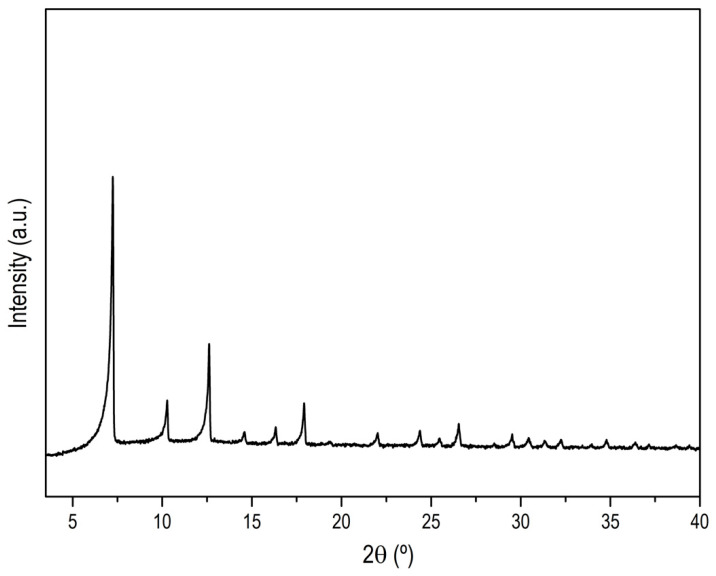
XRD pattern of ZIF-67.

**Figure A2:**
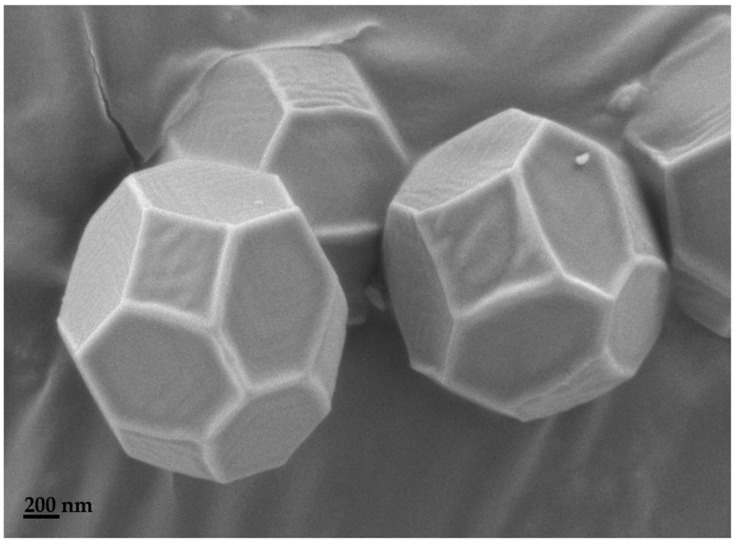
SEM micrograph of ZIF-67.

**Figure A3:**
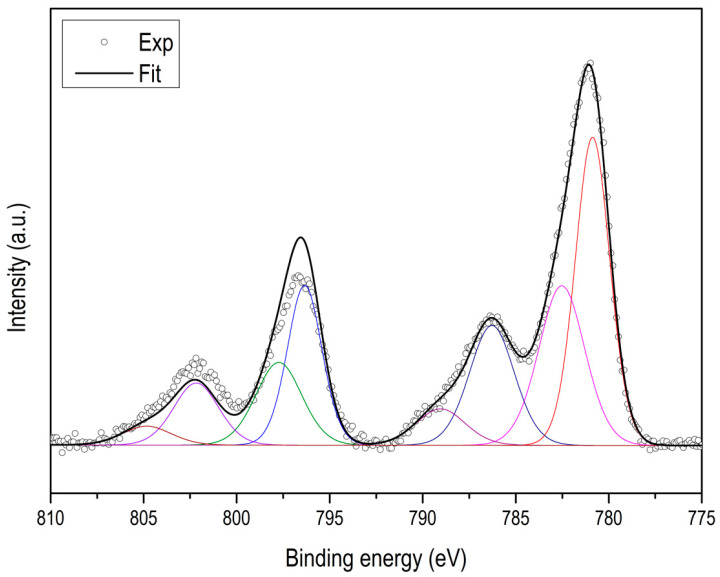
XPS spectra of ZIF-67 where it is clearly seen that it corresponds to Co^2+^.

**Figure A4:**
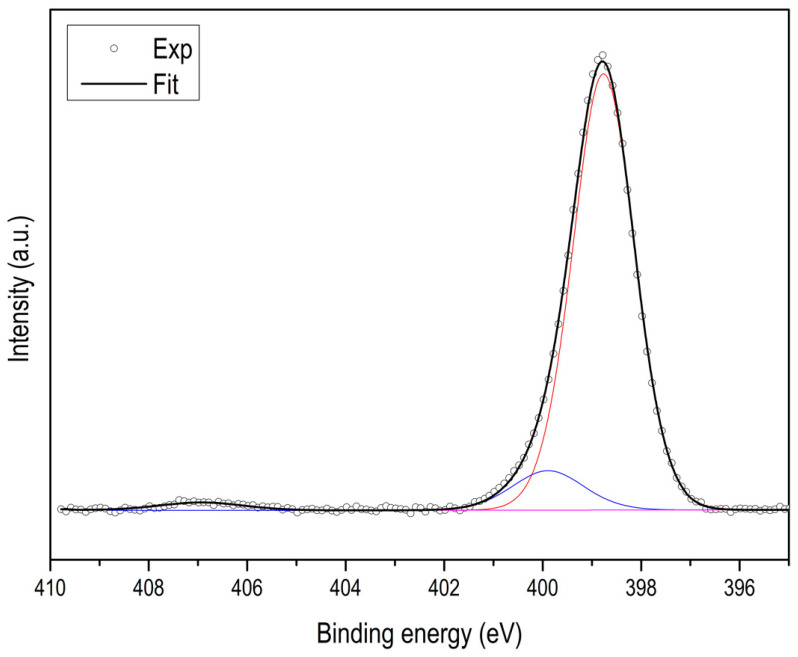
N XPS spectra of ZIF-67.

**Figure A5:**
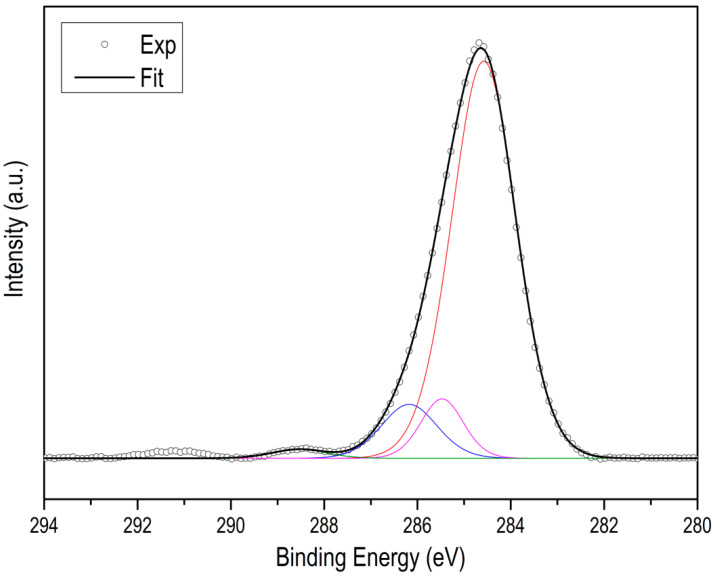
C XPS spectra of ZIF-67.

Figure A1, Figure A2, Figure A3, Figure A4 and Figure A5 show the characterization of ZIF-67 (ZIF-8 is not shown because it is practically the same) where it is clearly shown that a crystalline material with large and very well-defined crystals has been obtained. Figure 1 shows the thermogram of the ZIF-67 in Ar atmosphere, where a single weight loss is observed around 600 °C, where the great stability of this MOF is evidenced. That weight loss corresponds to the evolution of two characteristic *m*/*z* (53, 82) of ZIF-67 together with the evolution of N_2_. Figure 2 shows the thermogram in an oxidizing atmosphere of the material obtained from the previous thermogravimetry. A weight loss around 270 °C can be observed, which corresponds to the evolution of CO_2_, NO and NO_2_, it is also observed that the evolution of NO is prolonged in time, which is indicative that there are different nitrogenous species. In order to determine the C/N ratio in the carbon phase, a calibration with calcium oxalate has been carried out where the ratio is approximately 3. Based on these data, different activated carbons have been prepared. The main results are collected in Table 1 and Figure 3.

**Figure A6:**
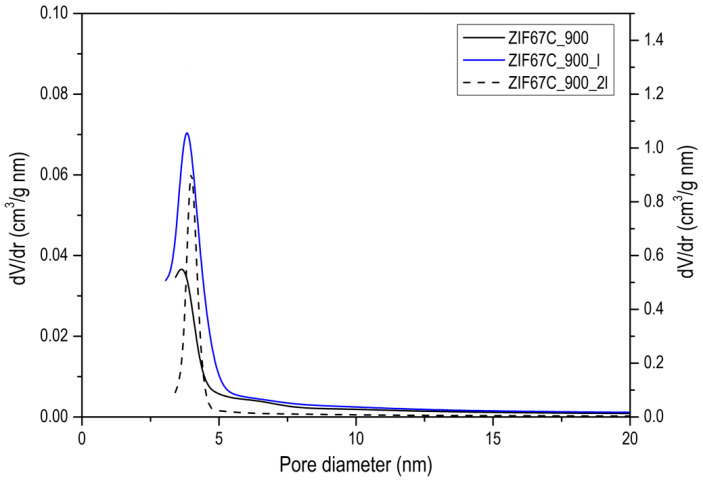
Pore size distribution of a series of activated carbons, where the one with the dashed line is represented following data from the right axis, while the rest go by the left one.

The evolution of activated carbons obtained from ZIF-8 and ZIF-67 show different trends. The most remarkable fact is the evolution of the surface area in the case of Zn, since what is observed is that in the treatment at 600 °C the area is about 1000 m^2^/g and a type I isotherm (see Figure 3), that reminds us of the original ZIF-8 (see [13]). However, after washing, it is observed that the area drops to 50 m^2^/g, which indicates that the carbon phase has not yet been produced, or it has been partially produced, and it has simply been a collapse of the ZIF-8 structure. In general, the carbons obtained from ZIF-8 show a greater development of porosity compared with ZIF-67. This is logical since Zn^2+^ is the most active catalyst in the development of porosity in activated carbons, in what is called chemical activation [21,22,23]. These authors found that this activating agent considerably increases the microporosity and up to a much lesser extent, mesoporosity when the temperature of the treatment is increased, as in our case, where it is observed that as the carbonization temperature increases, porosity develops, especially in the opening of the elbow in the adsorption isotherm. It is also important to point out that even at temperatures of 900 °C, the Zn has not evaporated as might be expected. In the acid treatment, part of the metal is eliminated, increasing the porosity, which is what the metal had blocked, observing a certain hysteresis. In the case of carbon materials obtained from ZIF-67, the evolution of porosity with pyrolysis temperature is clearly seen, but perhaps the most remarkable thing is the change in porosity with washing, making one or two washing steps to increase porosity by up to 70% (see Figure 3b). Another aspect to highlight is the fact that between 700 and 900 °C, the increase in area after 2 washes is very small. Which indicates that cobalt is not a good activating agent. When we go up to 1000 °C a decrease in the area is observed due to the “graphitization” process. In order to better characterize the porosity of the material, an analysis of the porosity distribution has been made using the Barrett-Joyner-Halenda (BJH) model, which is exclusive to mesoporosity [24]. It has been performed on the heat-treated ZIF-67 shown in Figure 3b and collected in Figure A6. Regarding the distribution of porosity, we see that it is centered on the border between microporosity and mesoporosity in the 3 cases, where the only relevant fact is that due to the shape of the curve, microporosity has developed somewhat more, as it has been commented previously.

**Figure A7:**
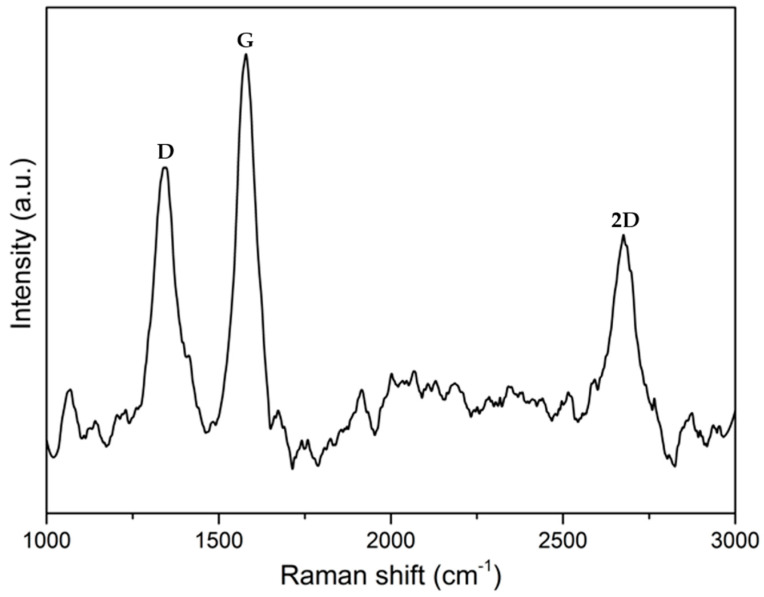
Raman spectra of ZIF67C_1000.

**Figure A8:**
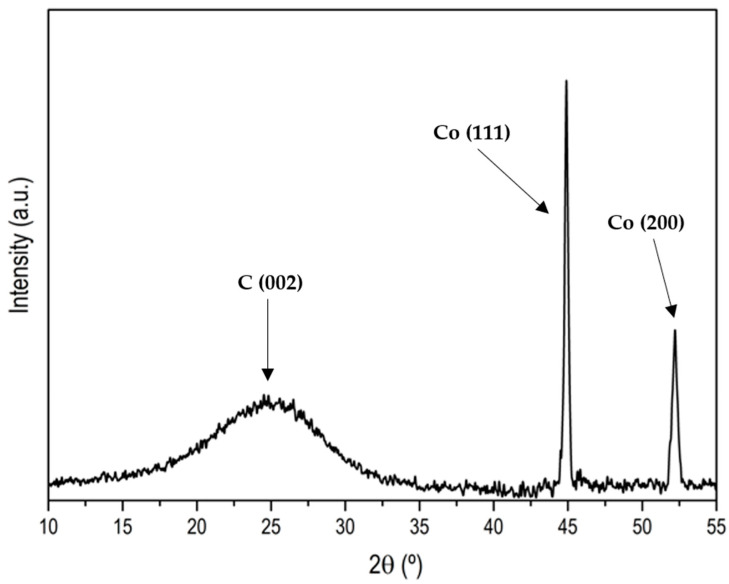
XRD pattern of ZIF67C_1000.

It is also noteworthy that in this case the ZIF-67 carbon material is more mesoporous than in the previous case, but above all the presence of a hysteresis loop is more notorious than in the case of Zn, so we can say that it is a type IV isotherm [24]. It is also remarkable that with the treatments carried out it is impossible to eliminate all cobalt. To eliminate it completely, some authors have used concentrated HF, which is much more aggressive [25]. The problem is they also found an ostensible modification of the surface chemistry of the material, with the generation of many nitro groups. Professor Gascón indicates that part of the Co cannot be eliminated as an advantage since they are very active catalysts [16]. Another difference between Zn and Co is that Co is a graphitization catalyst [26], so carbons obtained at 900–1000 °C will be much more ordered, as it can be seen in the X-ray diffractogram and in the Raman spectroscopy. Figure A7 and Figure A8 show the Raman and XRD spectra of ZIF-67 treated at 1000 °C. The spectrum shows two main bands that are called G and D (1580 cm^−1^, 1350 cm^−1^) that are related to sp^2^ graphitic carbon, and to disordered carbon or defects, respectively. The relationship between the bands G/D increase with the treatment temperature, in addition to the temperature of 1000 °C the band at 2700 cm^−1^ is perfectly visible, while at 900 °C it is only visible, this band is associated with 2D graphitic carbon, also called graphene band [27]. The X-ray diffractogram shows a series of peaks associated with “graphite” (plane (002), JCPDS No 75-1621) and metallic cobalt (plane (111), plane (200), JCPDS No 15-0806). There is no presence of carbides, nitrides or cobalt oxides observed. It is noteworthy that with increasing temperature the peaks become sharper, as one would expect. In the case that the precursor is ZIF-8, it is noteworthy that the peak associated with graphite is rather a broad band, and in this case, ZnO peaks are observed, and not only Zn. The Raman spectrum of ZIF-8 does not show the band at 2700 cm−1 that the ratio between the G/D band is always below 1, which once again highlights the non-graphitic character of this carbon material.

**Figure 4:**
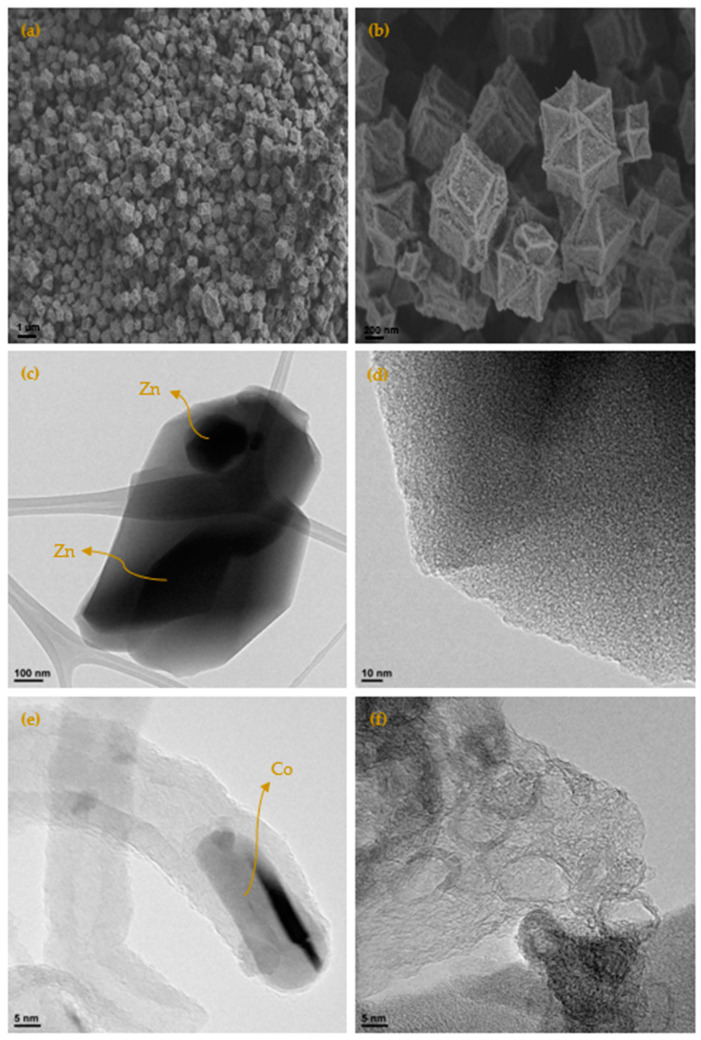
(**a**) SEM micrograph by means of secondary electrons of the carbonized sample at 700 °C (ZIF67C_700); (**b**) detail of the micrograph; (**c**) TEM micrograph of the sample ZIF8C_700, where the turbostratic microstructure of the carbon and the Zn particle can be clearly seen; (**d**) detail of the micrograph of the turbostratic zone; (**e**) TEM micrograph of the sample ZIF67C_900, where the formation of a carbon nanotube is shown, with the cobalt particle at one end and the highly ordered carbonaceous structure; (**f**) TEM micrograph of sample ZIF67C_900_2l, where it is clearly seen that the cobalt particles have been removed leaving the very crystalline structure of the carbon phase.

**Figure A9:**
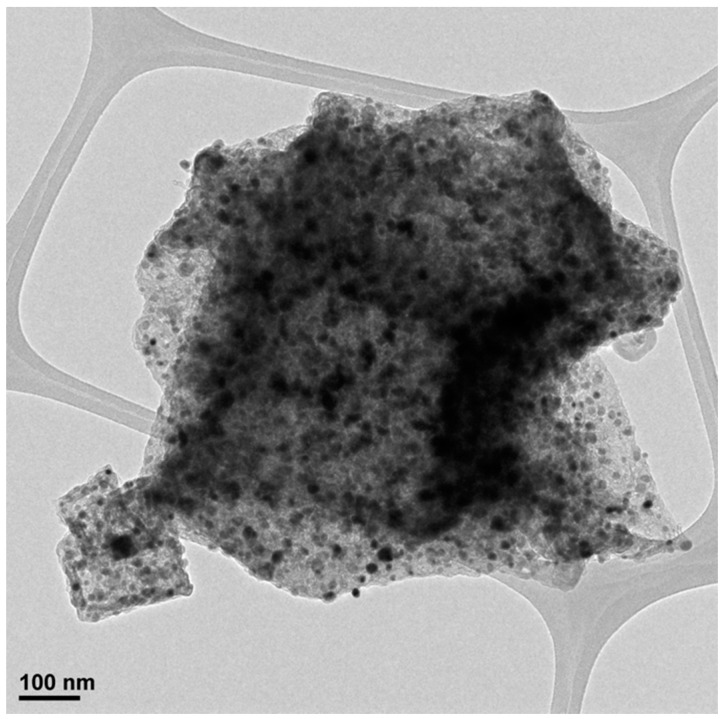
TEM micrograph of ZIF-67 carbonized at 700 °C.

**Figure A10:**
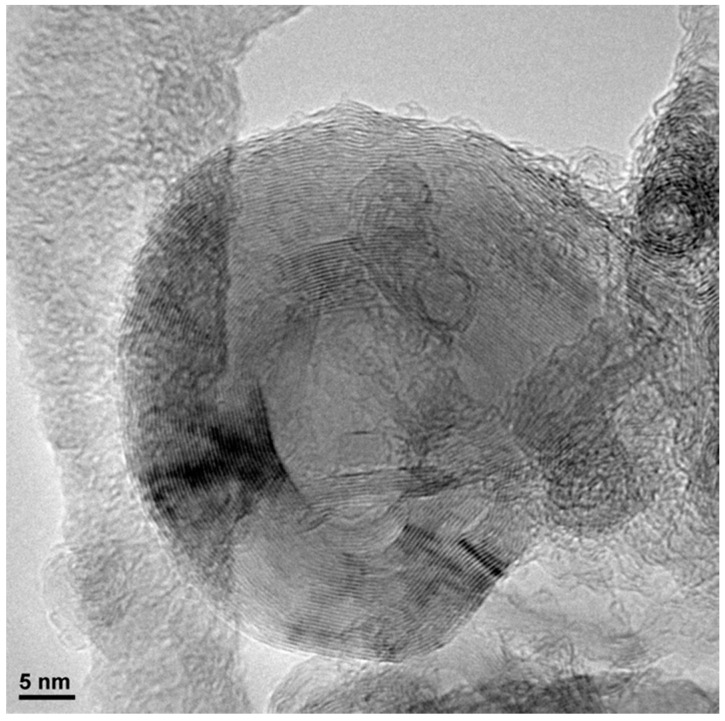
TEM micrograph of ZIF-67 carbonized at 900 °C and washed two times.

Figure 4 show the micrographs of the different carbons obtained at 700 °C (more micrographs may be found in the Appendix A, (see Figure A9 and Figure A10), where it is clearly seen that it maintains the original shape of the ZIF-67. It is as if the ZIF had undergone contraction. From 900 °C, the appearance of carbon whiskers is observed, probably due to the formation of CH_4_ in the carbonization process that decomposes on the cobalt nanoparticles forming graphitic carbon and hydrogen [28,29]. The TEM micrographs show in great detail the formation of these structures where the carbon is highly ordered. Such ordered structures linked to cobalt are also observed in other areas. It is especially revealing when the carbon material is treated with HCl, in which a large part of the cobalt has been extracted, observing the encapsulated and graphitic zones. Although the cobalt content is in the order of 5 wt.%, practically no nanoparticles are observed, which highlights what was previously mentioned that the cobalt is either in small clusters or monoatomically dispersed, linked to the carbon material through a nitrogen bond. The appearance of these cavities is linked to the development of the observed porosity. The activated carbons obtained from both ZIF show a great variety of porosity that goes from microporous to mesoporous, although the total area is not exceptional. In addition to this fact the presence of cobalt could be very useful in some applications. Furthermore, the high content of nitrogen that some carbons have, greater than 15%, is remarkable. The appearance of these cavities is linked to the development of the observed porosity. The TEM micrographs of the ZIF8C_700 sample are shown in the same figure where an activated carbon particle is clearly observed which contains a large Zn particle, the microstructure of the carbon phase is also shown, where it is clearly seen that it is turbostratic, as expected, and reconfirms what was previously obtained by Raman and XRD spectroscopy: only cobalt generates ordered structures in the carbon phase.

As we have previously commented, a key aspect is the nitrogen content. This is shown in Table 2. It is noteworthy that the content is 25% in the case of carbon obtained at 600 °C and this decreases greatly as a function of carbonization temperature independently from the ZIF-precursor. Although not only the nitrogen content is important, but also the type of nitrogen, since not all present the same interaction with CO_2_ and its catalytic activity in electrochemistry, either as supercapacitor or as electrodes in ORR reactions [30,31].

**Figure 5:**
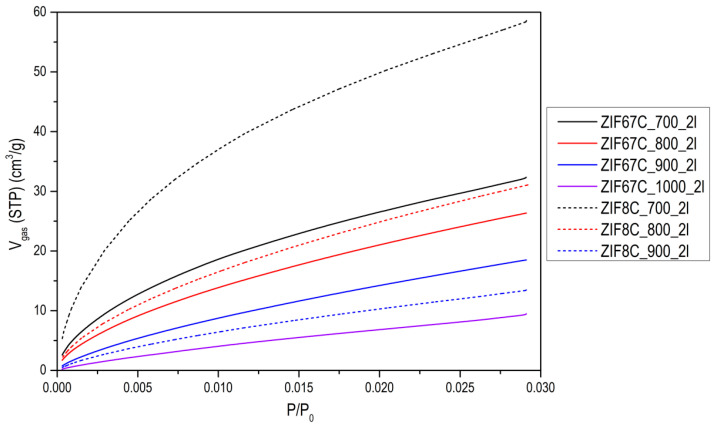
CO_2_ adsorption isotherms at 0 °C of some of the activated carbons.

**Figure A11:**
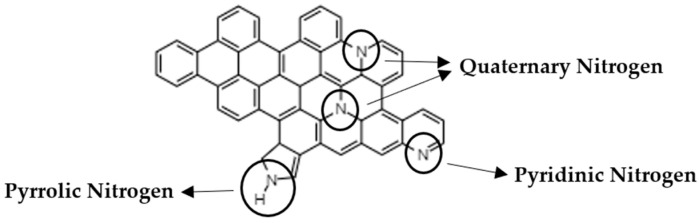
Schematic drawing of different groups of N in a graphene sheet.

Using XPS, it is possible to determine the type and quantity of nitrogen species. As an example, two spectra are shown corresponding to ZIF-67 carbonized at 600 °C and at 900 °C (see Figure 5), in both cases the N 1s signal can be deconvolved into three types of nitrogen species with binding energies of 398.7 eV, 400.3 eV and 401.5 eV, which can be attributed to pyridine, pyrrole and quaternary nitrogen, respectively (see Figure A11). Table 2 collects all the results, where we can highlight that not only the total nitrogen decreases, but also that the relationship between them changes ostensibly. The most remarkable thing is that the quaternary nitrogen content decreases very little, while the pyridine and pyrrolic nitrogen decrease much more.

**Figure A12:**
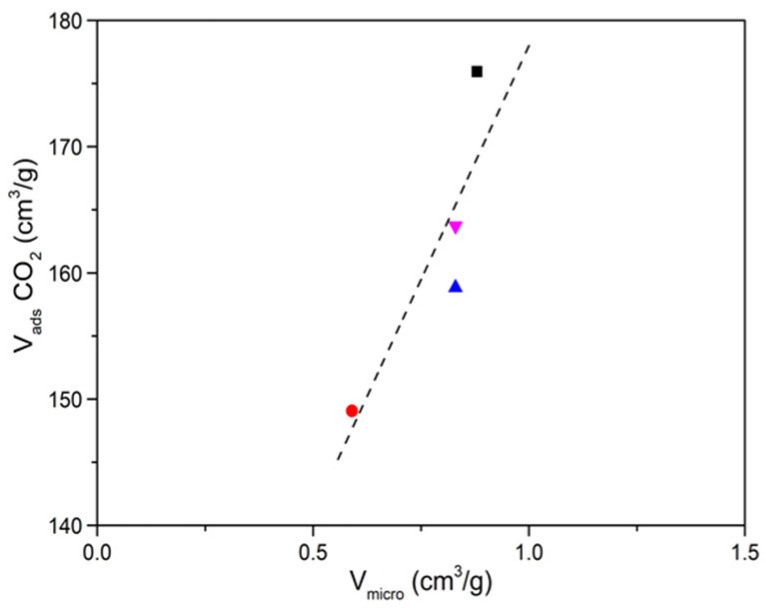
Relationship between microporous volume and adsorption capacity of CO_2_ (0 °C, 1 bar). 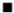
—Reference [29]; 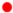
—Reference [30]; 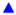
—Reference [31]; 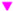
—Reference [32].

The CO_2_ adsorption isotherms (see Figure 4) are clearly revealing, where it can be seen that with the increase in carbonization temperature the CO_2_ adsorption capacity decreases, this occurs for both precursors. As previously mentioned, with the increase in carbonization temperature, the surface area and the volume of micropores increase, mainly in carbon obtained from ZIF-8, this fact seems to contradict what was previously published where they find a close relationship between the volume of micropores and the adsorbed CO_2_ (see Figure A12 of the Appendix A) [32,33,34,35]. However, the nitrogen groups that these carbons have must be taken into account, which at low temperatures are really high. Regardless of the type of nitrogenous group, their presence increases the basicity of the carbon framework which in turn will anchor the electron deficient carbon of CO_2_ to the carbon pore surface by Lewis acid-base (N atom) interactions [36]. Therefore, the nitrogen in the surface groups provides the necessary pair of electrons that will generate an attractive site to adsorb the electron-deficient carbon of the CO_2_ molecule. In our case we have 3 types of nitrogen: pyridine, pyrrolic and quaternary. The first two decrease to a greater extent than the latter, with the increase in carbonization temperature, so they must be the main responsible for the adsorption of CO_2_.

In the case of the pyrrolic group, both H and N interact with CO_2_, despite the research carried out by Lim [37], showing that the main interaction is between the oxygen of the CO_2_ molecule and H, these authors claim that this group is the main responsible for the adsorption of CO_2_. However, the pyridine groups are the ones that contribute with the most basic character to the carbon, therefore they should greatly increase the adsorption capacity. This statement is true if we assume that CO_2_ is adsorbed through the C molecule in all cases, but as we have mentioned in the case of pyrrolic it is through the interaction of O with H, which is much more favorable. Kretzschmar and co. [38] have developed a series of active carbons, where in a range of temperatures the volume of micropores is practically the same, such as the carbon obtained from ZIF-67 in our research, finds a great difference in the adsorption of CO_2_, and it depends mainly on the pyrrolic groups, since in these carbons practically generates quaternary N and pyrrolic groups.

More recently, a very extensive theoretical study has been carried out by Gholami et al. [39], in which they show that due to the strong interaction between the pyrrole group and CO_2_ it is possible to double the CO_2_ adsorption capacity. What once again highlights what was said above about the fact that the main species responsible for the increase in adsorption is the pyrrolic group which corroborate our results. These authors comment that a simple way to see the interaction is through the Freundlich isotherm [40].

θ = KP^1/n^ (1)

where θ is the fractional coverage, P is the partial pressure of the adsorbate, and K and n are both empirical constants which indicate adsorption capacity and adsorption intensity, respectively. Where n is a parameter greater than one, if it is one it would be Henry’s law which would imply that there is no interaction. The larger n is, the greater the interaction between the support and the adsorbate. In our case, this parameter ranges between 2 for the one with the highest nitrogen content and 1.33 for the one with the lowest nitrogen content.

## 4. Conclusions

In the present investigation, a series of activated carbons have been developed using MOFs (ZIF-8, ZIF-67) as precursors. The most notable aspect is that carbons with a N content greater than 20 at.% have been achieved. Nitrogen content in activated carbon decreases with increasing treatment temperature. The CO_2_ adsorption capacity is very high. The adsorption capacity depends on the micropore volume and the N content. Nitrogen is mainly found forming pyridine, pyrrolic structures and as quaternary nitrogen. The first two are responsible for the high adsorption capacity of CO_2_.

The presence of Zn and Co generates carbons with different microstructures. Cobalt (ZIF-67) generates graphitic structures, zinc (ZIF-8) generates turbostratic structures. Zn generates carbons with a more developed porosity.

The activated carbons generated from ZIF-8 have a higher porosity and a slightly higher nitrogen content, which makes them ideal for the selective adsorption of CO_2_.

The activated carbons developed from ZIF-67 are more graphitic, together with the fact that they have a high N content, making them ideal for use in electrochemistry.

## Figures and Tables

**Table 1:** Textural properties from the obtained carbons.

Sample	BET (m^2^/g)	Micropore Volume (cm^3^/g)	Pore Volume (cm^3^/g)	V_Mesopore_/V_Total_ × 100 (%)
ZIF8C_600	1000	0.51	0.56	9
ZIF8C_600_2l	50	0.03	0.05	40
ZIF8C_700	336	0.16	0.22	27
ZIF8C_700_2l	656	0.33	0.39	15
ZIF8C_800	616	0.30	0.37	19
ZIF8C_800_2l	862	0.42	0.55	24
ZIF8C_900	775	0.40	0.49	18
ZIF8C_900_2l	1022	0.49	0.66	26
ZIF67C_600	255	0.10	0.19	47
ZIF67C_600_2l	300	0.12	0.22	45
ZIF67C_700	298	0.11	0.23	52
ZIF67C_700_2l	457	0.16	0.39	59
ZIF67C_800	300	0.10	0.22	54
ZIF67C_800_2l	475	0.17	0.41	58
ZIF67C_900	282	0.09	0.20	55
ZIF67C_900_2l	486	0.16	0.37	57
ZIF67C_1000	189	0.07	0.23	69
ZIF67C_1000_2l	250	0.12	0.25	52

**Table 2:** Summary of the XPS results with classification of N into its expected bond types.

Sample	N_Total_ (at.%)	N_Pyridinic_ (at.%)	N_Pyrrolic_ (at.%)	N_Quaternary_ (at.%)
ZIF8C_600_2l	25	17.6	6.6	0.8
ZIF8C_700_2l	19.0	9.9	8.1	1
ZIF8C_800_2l	11.5	7.2	3.4	0.9
ZIF8C_900_2l	4.0	3.1	0.2	0.7
ZIF67C_600_2l	20.5	13	6	1
ZIF67C_700_2l	17.3	9.6	7,3	1.4
ZIF67C_800_2l	11.2	7.1	3.2	0.9
ZIF67C_900_2l	5.2	4.0	0.4	0.8

## Appendix A

In this appendix, the additional figures mentioned in the text are presented, which are not essential but helping the understanding of what’s said in the main body of the article.
